# Supplementary material for: Blood Serum Stimulates the Virulence Potential of Mucorales through Enhancement in Mitochondrial Oxidative Metabolism and Rhizoferrin Production
Source: J Fungi (Basel). 2023 Nov 22;9(12):1127. doi: 10.3390/jof9121127 (PMC10744254; doi:10.3390/jof9121127)
Supplement: Supplementary file 1 [file jof-09-01127-s001.zip › jof-2667981-supplementary.pdf]

Table S1. Strains used in this study

Strain	Genotype	Parental strain	Source
NRRL3631 (avirulent control)	WT	*M. lusitanicus* (+)	
R7B (virulent control)	WT, (*leuA*$^-$)	*M. lusitanicus* CBS 277.49 (-)	(19)
MU636	WT, (*leuA*$^-$, *pyrG*$^+$)	MU402	(20)
M5	(*leuA*$^-$, *adh1*$^-$)	R7B	(12)
Δ*arl2*	(*leuA*$^-$, *pyrG*$^+$, *arl2*$^-$)	MU402	(15)
Δ*rfs*	(*leuA*$^-$, *pyrG*$^+$, *rfs*$^-$)	MU402	(13)
Rhizopus arrhizus (RA12)	WT		Clinical isolated kindly provided by Dr. Urlike Binder
Mucor circinelloides (MC39)	WT		Clinical isolated kindly provided by Dr. Urlike Binder
Mucor racemosus (MR11)	WT		Clinical isolated kindly provided by Dr. Urlike Binder
Lichtheimia corymbifera (LC42)	WT		Clinical isolated kindly provided by Dr. Urlike Binder
Candida albicans	WT		Clinical isolated from Hospital Civil, Morelia, Michoacán, México.

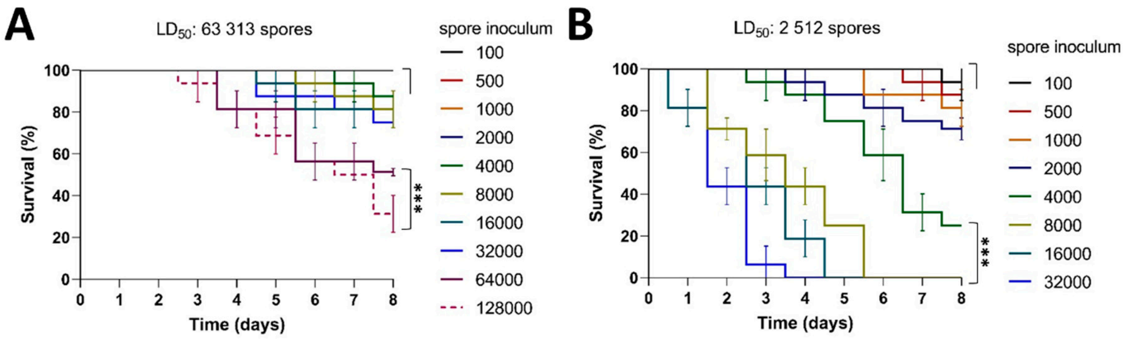

Figure S1. Effect of the inoculum size of *M. lusitanicus* and *R. arrhizus* on the survival of *G. mellonella*. Spores from **A)** *M. lusitanicus* (MU636) and **B)** *R. arrhizus* (RA12) were used to infect *G. mellonella*. After the larvae were infected with the spores, the survival was monitored daily for a period of 8 days. The lethal dose 50 (LD_{50}) was calculated. Data were statistically analyzed using the Mantel-Cox test ***, $P<0.01$. The results presented are the average values obtained from four independent experiments.

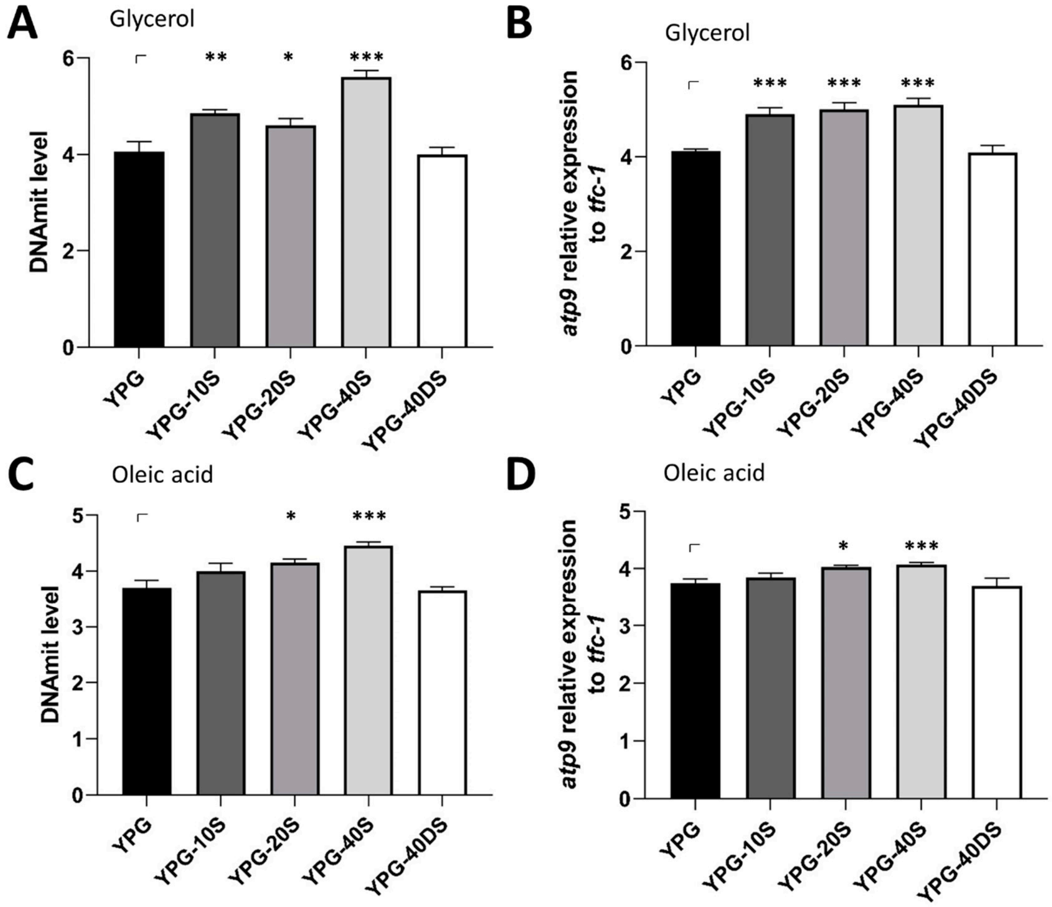

Figure S2. Effect of blood serum on the mitochondrial content during aerobic germination of *M. lusitanicus* in non-fermentable carbon sources. Spores from the R7B strain produced on YPG supplemented or not with 10% (YPG-10S), 20% (YPG-20S), and 40% (YPG-40S) of native blood serum or 40% of denatured blood serum (YPG-40DS) were aerobically germinated for 4 h in YNB supplemented with 111 mM glycerol or 111 mM oleic acid. **A, C)** qPCR analysis using total DNA isolated from mycelium of 4 h was performed to quantify the mitochondrial gene *atp9.* **B, D)** Total ARN from mycelium after 4 h of growth was isolated for quantification of the expression of the molecular marker of mitochondrial activity, *atp9*. The data were statistically analyzed using the ANOVA and Fisher´s exact test. *P < 0.05, **P < 0.01, ***P < 0.001.

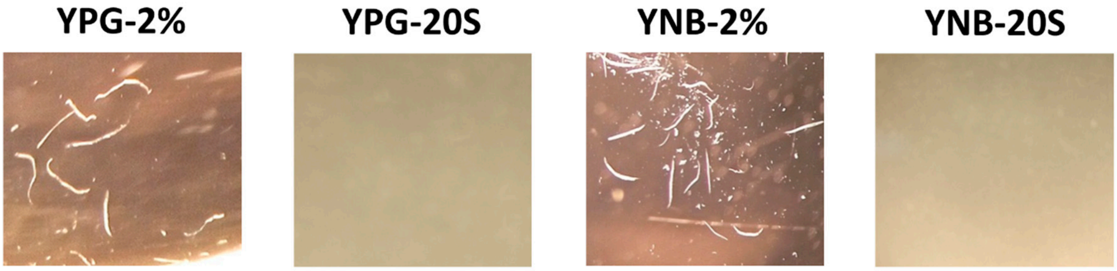

Figure S3. Effect of the addition of blood serum to culture broth on the observation of the nematodes under the stereoscope. YPG-2%, YPG-2% supplemented with 20% of blood serum (YPG-20S), YNB-2%, and YNB-% supplemented with 20% of blood serum (YNB-20S). Photos were taken using 10x optical microscope.

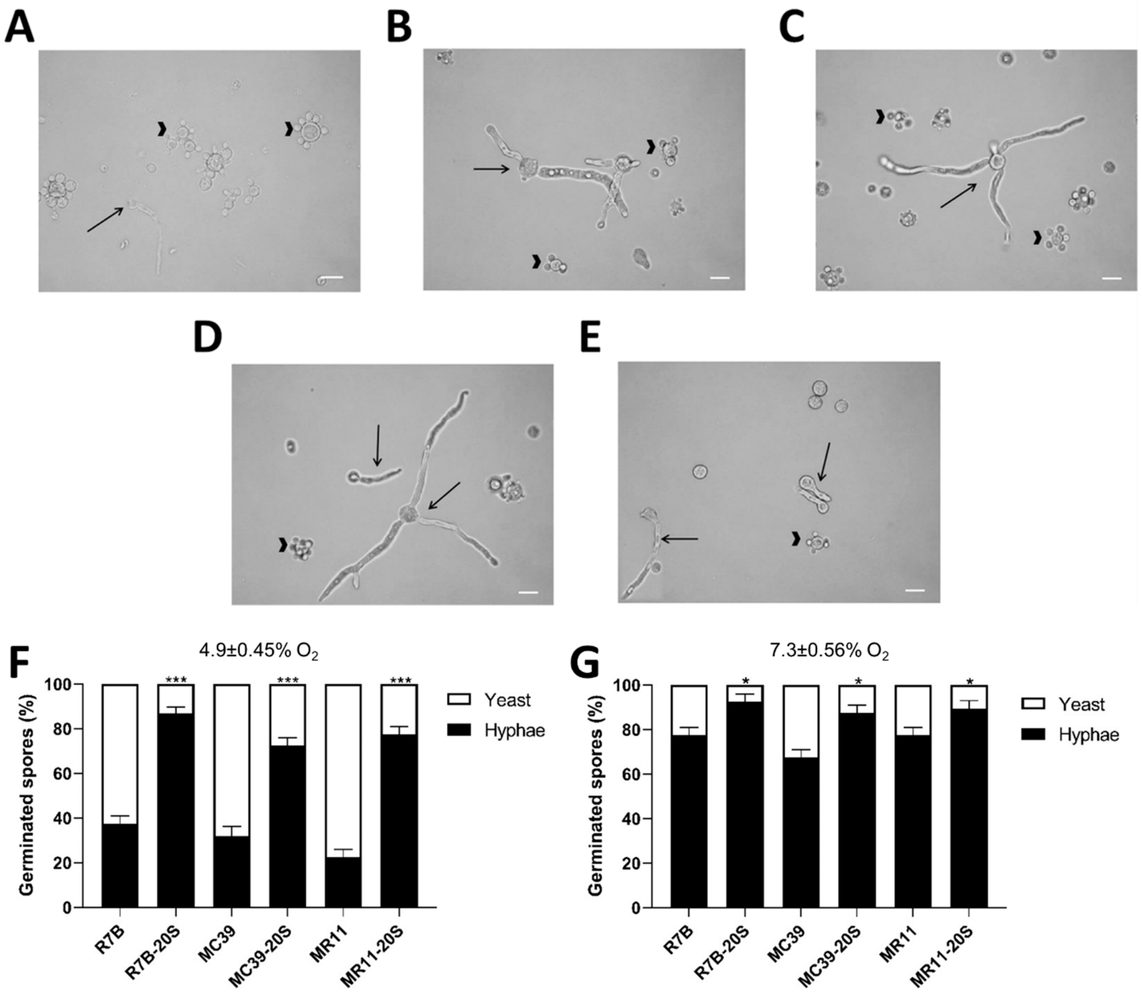

Figure S4. Effects of blood serum on the germination of *M. lusitanicus* after 6 h of growth under low oxygen levels (LOL). Representative photographs of the morphology under low oxygen levels of **A)** YPG, **B)** YPG-10S, **C)** YPG-20S, **D)** YPG-40S, and **E)** YPG-40DS (-S, native blood serum. -DS, denatured blood serum). The arrows represent the germlings and the arrow head represents the yeast cells. Bar = 20 µm. **F, G)** Spores from YPG or YPG supplemented with blood serum (-20S) of *M. lusitanicus* (R7B), *M. circinelloides* (MC39) or *M. racemosus* (MR11) were germinated under low oxygen levels (4.9 ± 0.45% of oxygen; 7.3 ± 0.56% of oxygen). The data were statistically analyzed using the ANOVA and Fisher´s exact test. *P < 0.05, ***P < 0.001.

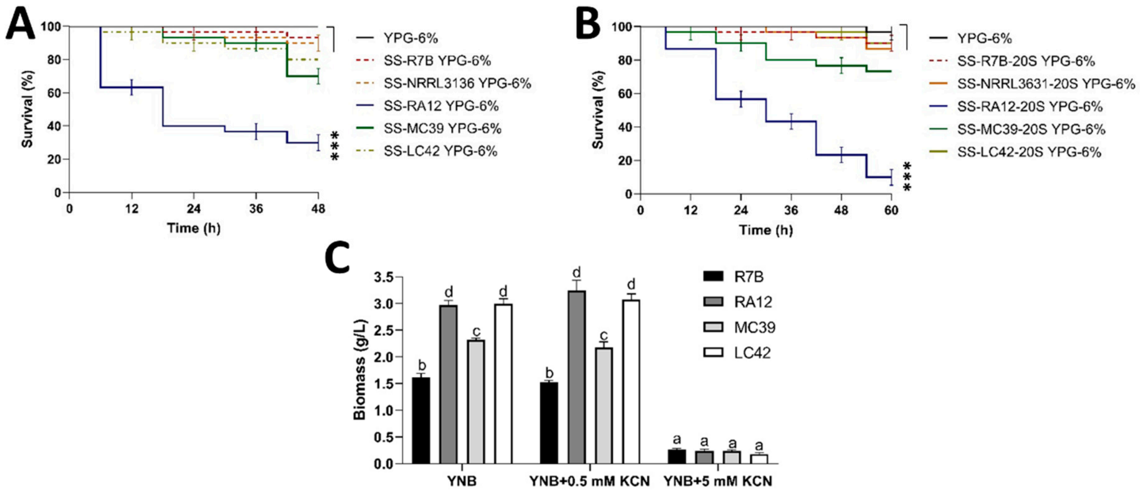

Figure S5. Effect of the toxicity of the SS from growth cultures of Mucorales under high glucose levels (6%) and KCN. Spores from *R. arrhizus*, *M. lusitanicus* (R7B and NRRL3136), *M. circinelloides* (MC39), and *L. corymbifera* (LC42) were produced on YPG or YPG supplemented with blood serum (-20S). The spores were grown in liquid YNB with 0.1% of glucose medium supplemented with **A)** 6% glucose (spores form YPG)**, B)** 6% glucose (spores form YPG-20S), YPG with **C)** 0.5 mM of KCN in order to obtain their SS after 48 h of growth under aerobic conditions and tested for their toxicity against *C. elegans*. A total of 15–20 nematodes were used per experiment and incubated at 18°C for 60 h. The results presented are the average values obtained from four independent experiments. Survival data were statistically analyzed using the Mantel-Cox test ***, $P<0.01$. Data were statistically analyzed using ANOVA with Fisher's exact test; the different letters shown statistical significance ($P < 0.001$).

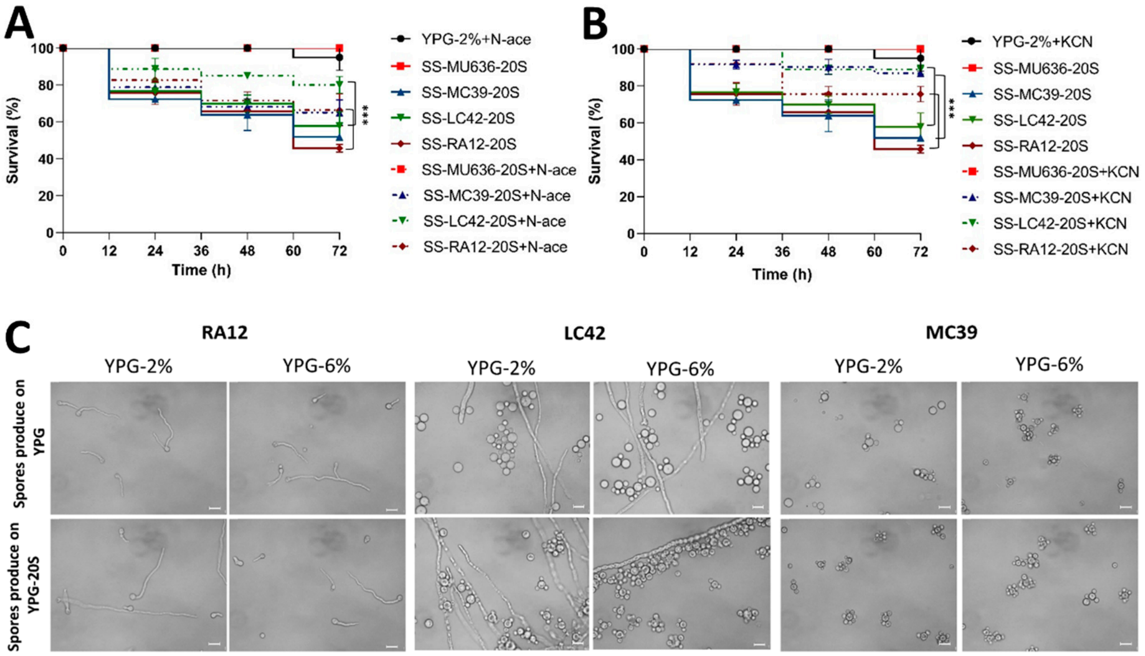

Figure S6.Effect of blood serum in SS toxicity and morphology during anaerobic germination of clinically relevant Mucorales. Spores from *R. arrhizus* (RA12), *M. lusitanicus* (MU636), *M. circinelloides* (MC39), and *L. corymbifera* (LC42) produced on YPG-20S were used to obtain their SSs from anaerobic growth in YPG-2% supplemented with **A)** 10 mM N-acetylcysteine (N-ace) and **B)** 0.5 mM KCN and tested for their toxicity against *C. elegans*. A total of 15–20 nematodes were used per experiment and incubated at 18°C for 72 h. **C)** Representative photographs from spores produced on YPG supplemented or not with blood serum of Mucorales germinating under anaerobic growth. The photographs were taken using a 40x optical microscope, bar = 20 μm. Survival data were statistically analyzed using the Mantel-Cox test ***, $P<0.01$. The results presented are the average values obtained from four independent experiments.

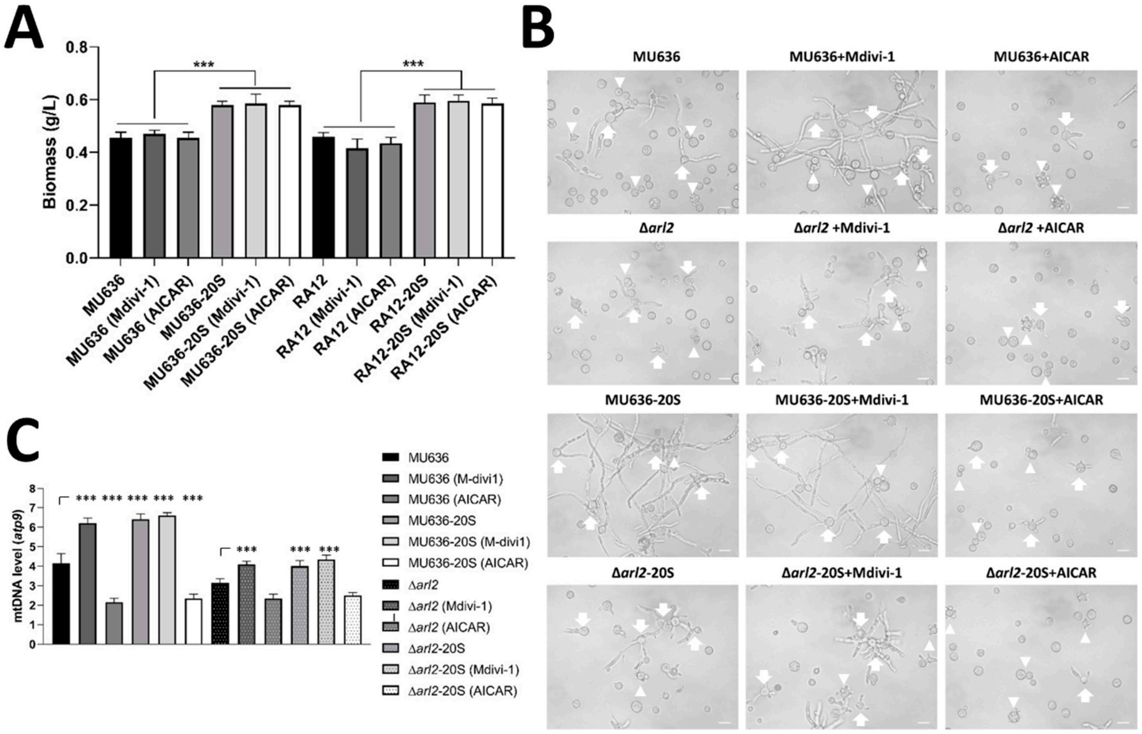

Figure S7. Effect of inducer AICAR and repressor Mdivi-1 of mitophagy in hyphal stimulation during growth at low oxygen levels in *M. lusitanicus* and *R. arrhizus*. A) Biomass was determined from the growth under aerobic conditions of spores produced in presence (-20S) or absence of blood serum from WT MU636 (*M. circinelloides*) and RA12 (*R. arrhizus*) during 48 h in liquid YNB-0.1% supplemented with 100 μM AICAR and 100 μM Mdivi-1. **B**) Representative photographs were taken from germinated spores produced in presence (-20S) or absence of blood serum from MU636 and Δ*arl2* strains from *M. lusitanicus* that were grown at low oxygen levels (4.9 ± 0.45% oxygen) during 6 h in liquid YPG-2% supplemented with 100 μM AICAR and 100 μM Mdivi-1 (scale bar = 20 μm). Hyphae are shown in white arrow and yeast in white arrow head. **C**) Total DNA was obtained from germinating spores after 6 h of growth under low oxygen levels from panel B, these geminating spores were used to quantified by qPCR the mitochondrial *atp9* gene using *tfc-1* as a genomic DNA marker. The results presented are the average values obtained from four independent experiments. The data were statistically analyzed using ANOVA and Fisher´s exact test. *** $P<0.001$.

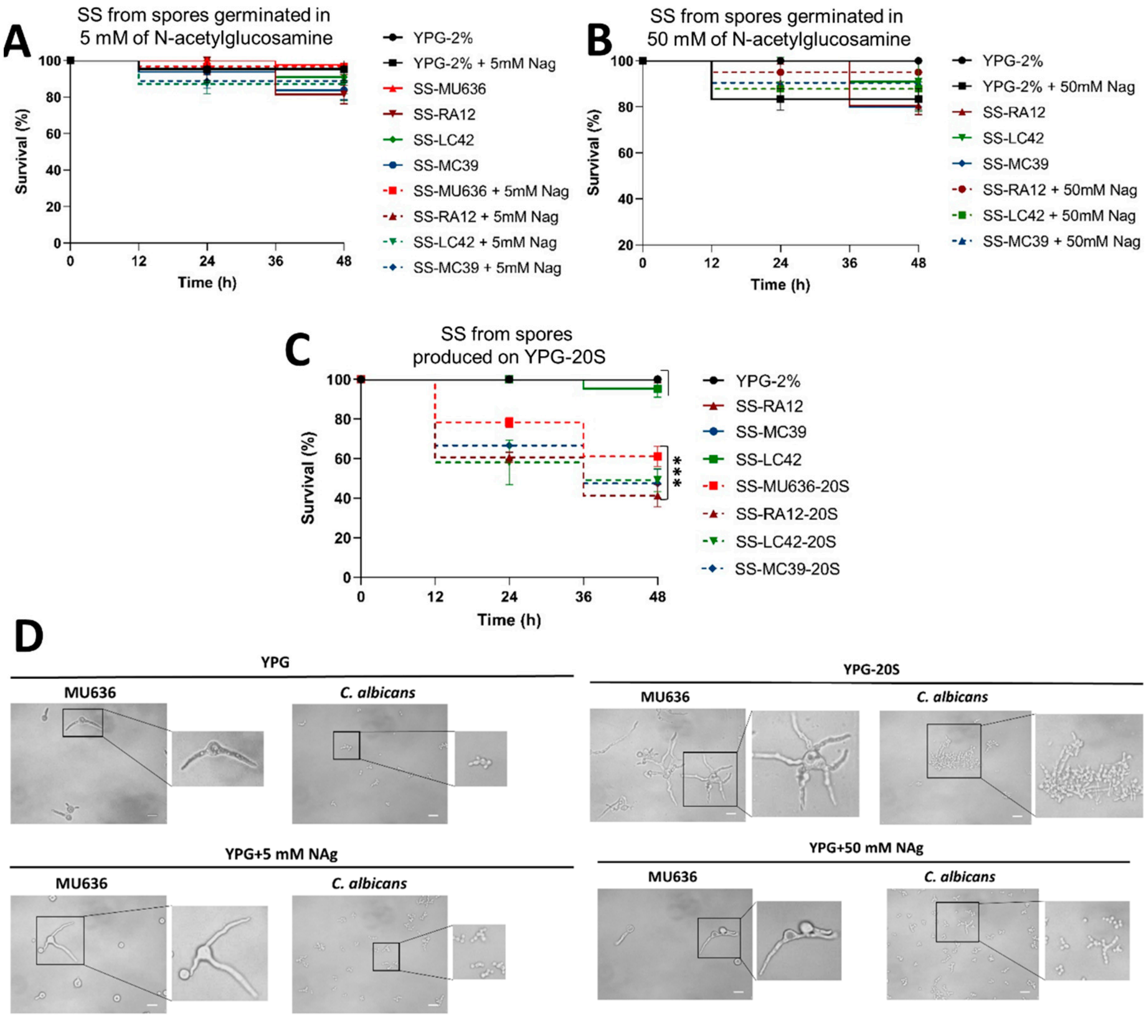

Figure S8. N-acetylglucosamine did not increase the toxicity of the SS from Mucorales. SSs obtained after 48 h of aerobic growth from the different Mucorales **A)** from spores that were germinated in YPG supplemented with 5 mM and **B)** 50 mM of N-acetylglucosamine, or **C)** SS obtained from the growth of spores that were produced on YPG supplemented with blood serum (YPG-20S) and assayed against *C. elegans*. A total of 15–20 nematodes were used per experiment and incubated at 18 °C for 48 h. **D)** Spores from MU636 or yeast cells of *C. albicans* germinated aerobically (150 rpm) in YPG in the presence of 5 or 50 mM of N-acetylglucosamine. Representative photographs at 4 h were taken using a 40x optical microscope, bar = 20 µm. Data were statistically analyzed using ANOVA with Fisher's exact test ***,

P < 0.001. Survival data were statistically analyzed using the Mantel–Cox test; ***, *P* < 0.01. The results presented are the average values obtained from four independent experiments.
